# Thrombotic Thrombocytopenic Purpura: What an Intensive Care Unit Doctor Needs to Know

**DOI:** 10.7759/cureus.7293

**Published:** 2020-03-16

**Authors:** Arian Bethencourt-Mirabal, Daylin Rodriguez Cesar, Dalie Ortet, Felix Hernandez, Gustavo Ferrer

**Affiliations:** 1 Pulmonary Critical Care, Aventura Hospital and Medical Center, Miami, USA; 2 Psychology, Penuel Counseling, Miami, USA; 3 Internal Medicine, Universidad Latina de Costa Rica, San Jose, CRI; 4 Internal Medicine, Aventura Hospital and Medical Center, Miami, USA; 5 Pulmonary Critical Care, Aventura Hospital and Medical Center, Aventura, USA

**Keywords:** peripheral blood, acute coronary

## Abstract

Accurate and prompt diagnoses of thrombotic microangiopathy (TMA) in the emergency room (ER) and intensive care unit (ICU) setting can be challenging since its presentation involve multiple organ systems, and comorbid diseases can be deceptive for an accurate diagnosis.

Here, we present the case of a patient, who upon arrival to the ER, reported severe chest pain radiating to his left shoulder, diaphoresis, headache, and nausea. Several numbers of small petechiae on the bilateral lower extremities were also found during physical examination. Laboratory data demonstrated elevated troponin levels, platelet count of 34, and hemoglobin of  8.7 g/l. Establishing a differential diagnosis between a microvascular occlusive disorder and acute coronary syndrome was imperative to reduce further clinical complications and mortality. A peripheral smear, which is an essential test in approaching the diagnosis of thrombotic thrombocytopenic purpura (TTP), was done and it identified an increased number of schistocytes. The laboratory findings narrowed the diagnosis to an immunological process, where the dysfunctional platelets caused coronary thrombosis and further intermittent coronary ischemia. In this case report, we discuss the atypical presentation of TTP, its differential diagnosis, and management in order to develop an effective treatment in the ER and ICU settings and to reduce the mortality rate.

## Introduction

Thrombotic thrombocytopenic purpura (TTP) is a relatively rare disorder whose hallmarks are thrombocytopenic, microangiopathic hemolytic anemia (MAHA), and neurological dysfunction. Atypical cases that included multiple organ system involvements have been reported in the literature. The diagnosis of TTP should be considered in any patient with unexplained thrombocytopenia and MAHA. In addition, TTP is the clinical manifestation of a heterogeneous group of underlying disorders driven by pathophysiology processes. The core laboratory features of TTP are those of MAHA (more than two schistocytes per oil immersion field on a peripheral smear, increased lactate dehydrogenase, and very low haptoglobin). Disintegrin and metalloproteinase with thrombospondin motifs (ADAMTS) activity is less than 10%. The differential diagnosis included several hematological and non-hematological disorders.

## Case presentation

The patient was a 56-year-old male who presented to the emergency room (ER) with complaints of chest pain, nausea, and headache. The pain was located on the left side of the chest, pressure like with radiation to the left shoulder and an intensity of 7 out of 10. Associated symptoms included diaphoresis and nausea. He also reported bleeding from his gums while brushing his teeth for the past seven days. Physical exam was remarkable for small petechial lesions in his lower extremities bilaterally. His complete blood count analysis showed evidence of thrombocytopenia, with a platelet count of 9 x 10 9/l and anemia, with a hemoglobin count of 8.7 g/l. Troponin was mildly elevated at 0.24 ng/ml; electrocardiogram (EKG) was unremarkable.

**Figure 1:**
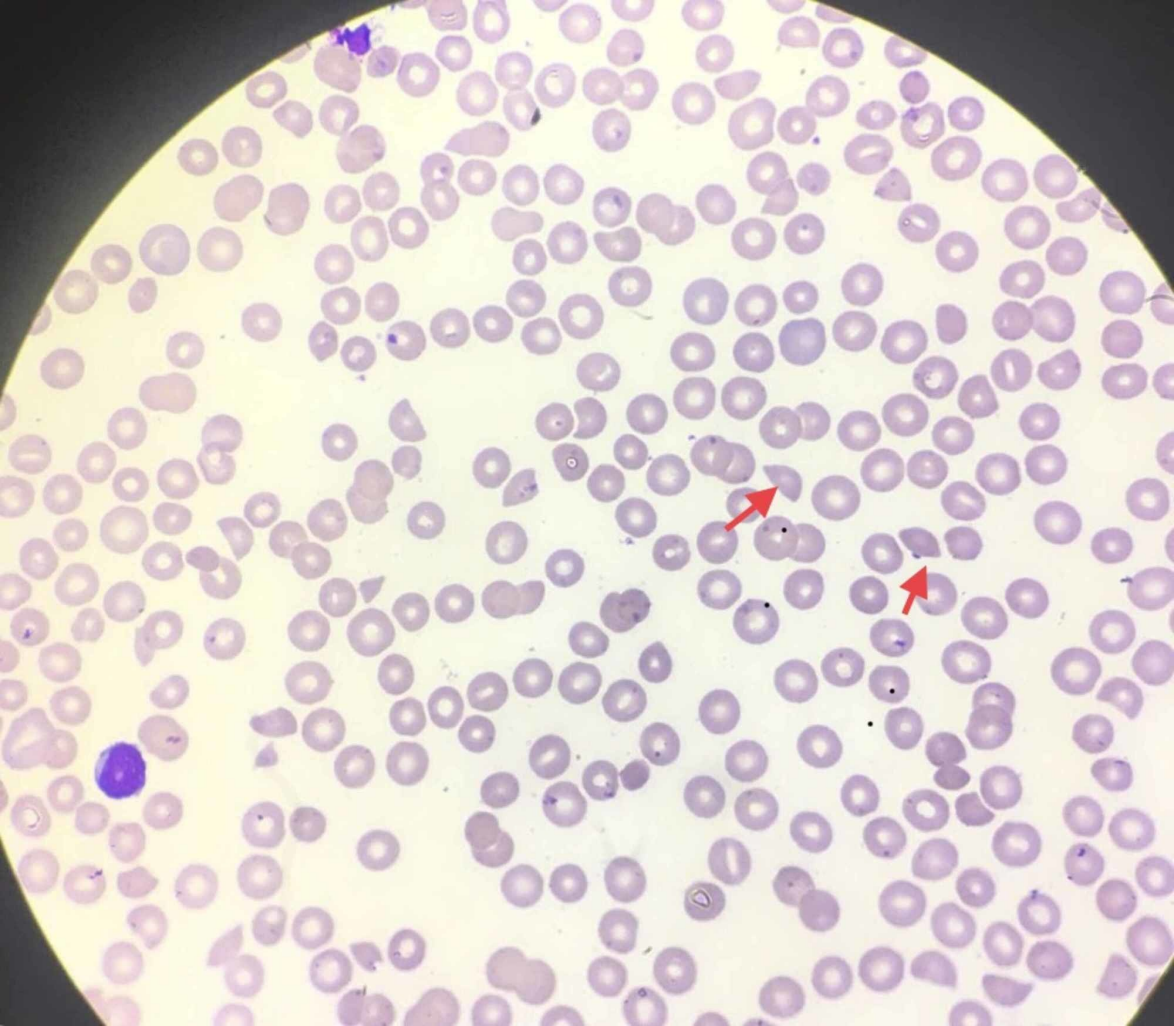
Peripheral smear of the patient; red arrows point to the schistocytes

At this point, based on the patient's presentation and his laboratory findings, the top differential diagnoses included acute coronary syndrome as well as an immunological reaction, such as TTP. A peripheral blood smear was performed which revealed the presence of more than 2% schistocytes (Figure 1). The presence of schistocytes was suggestive of thrombotic microangiopathy (TMA) and TTP was highly suspected. We suspected that abnormal platelet aggregation led to a pro-thrombotic state which ultimately led to intermittent coronary ischemia. To confirm the diagnosis, ADAMTS13 level was ordered; however, the test results usually take days to return as it is done outside our institution. Based on the clinical presentation and evidence present, we decided to treat the patient with a presumptive diagnosis of TTP. He received plasma exchange and after frothing for days, his platelet count returned to normal levels.

## Discussion

Epidemiology

In 1924, Moschcowitz described a case of a 16-year-old girl with weakness, pallor, purpura, and hemiparesis who died after 14 days with cardiac failure. Autopsy revealed hyaline thrombi in terminal arterioles and capillaries throughout most organs, including the kidney. This report was the first description of TTP and was also known as ADAMTS13 deficiency mediated [1]. In 1982, unusually large multicenter of von Willbrand factor was observed in patients with chronic and relapsing TTP. This previous finding led to the discovery of a von Willebrand factor-cleaving protease that was subsequently characterized as ADAMTS13 [1]. 

The incidence of acquired TTP is much greater in adults (2.9 cases per one million per year) than in children (0.1 cases per one million per year) [1]. Over a 25-year period in the Sacramento, California region (population at risk, 1.2 million), at least 176 documented cases of TTP were reported. In another one-year study, 20 institutions reported 115 patients with TTP. A French national registry found that the rate of TTP in France was 13 cases per million population [1]. Untreated, TTP has a mortality rate as high as 90%. With the plasma exchange, the mortality rated is reduced to 10%-20%. Acute morbidity includes ischemic events like strokes, transient ischemic attack, myocardial infarction, bleeding, and azotemia.

Pathophysiology

A deficiency of this enzyme can be either acquired or hereditary. Hereditary TTP (also called Upshaw-Schulman syndrome) is caused by homozygous or compound heterozygous ADAMTS13 mutation [2]. Acquired TTP it is an autoimmune disorder caused by autoantibody inhibition of ADAMTS13 activity.

The primary cause of TTP is a failure of the ADAMTS13 enzyme involved in blood clotting [3]. The failure of the enzyme leads to hyperactive blood coagulation. Thus, there is a resultant formation of blood clots in the small blood vessels. The formation of blood clots in these vessels is termed TTP. The clots can limit the free flow of oxygen-rich blood to other organs; hence, serious health problems can occur.

Inherited TTP is caused by a mutation of the ADAMTS13 gene, resulting in a corresponding enzyme failure. The cause of acquired TTP is distinct from inherited TTP. Acquired TTP is not caused by the failure of the ADAMTS13 gene; instead, it results from the body producing antibodies that block the ADAMTS13 gene from its regular and intended activity.

Clinical manifestations

Patients with TTP typically report acute or subacute onset of neurological symptoms. The cardinal symptoms are neurological manifestations which include alternate mental status, seizures, hemiplegia, visual disturbance, and aphasia as well as fatigue associated with anemia and petechia. Fever is reported in 50% of the cases. Some patients have minimal abnormalities while others are critically ill [4]. In the current case, the patient presented with chest pain due to coronary transient ischemia.

Diagnosis

The core laboratory features of TTP are those of MAHA (more than two schistocytes per oil immersion field on a peripheral smear, increased lactate dehydrogenase, and very low haptoglobin). ADAMTS activity is less than 10%. The differential diagnosis included several hematological and non-hematological disorders.

Treatment

Once the diagnosis of TTP is thought to be likely, therapy should be quick because of the proclivity of the disease to progress rapidly. All patients should be initially treated in the intensive care unit (ICU). Platelet transfusion should be avoided except in cases of life-threatening bleeding. TTP is typically treated using plasma therapy, steroids, rituximab or newer drugs. According to Kuter et al. (2010), plasmapheresis remains the primary method to treat TTP [5,6]. Plasmapheresis is performed daily until symptoms, such as renal failure and abdominal pain, are reduced and platelet count returns to a normal level from intravascular hemolysis. If plasma exchange cannot be initiated in a timely manner, an infusion of 30 mL/kg of fresh frozen plasma (FFP) daily can be a temporizing maneuver [6].

Steroids are effective in decreasing inflammation and reducing the activity of the immune system; a higher than the standard dose of steroids is recommended, particularly for patients who are new to treatment [7]. Rituximab is an antibody used in conjunction with plasma therapy; however, it is not without side effects, which in some cases can be fatal.

Newer drugs have been discovered. N-acetylcysteine (NAC) is one such drug that inhibits platelets from linking with vWF, which promotes clotting. Bortezomib prevents plasma cell depletion; thus, there may be no need to conduct plasma therapy. Caplacizumab is the first only FDA-approve therapy for adults with acquired TTP in combination with plasma exchange and immunosuppressive therapy. Scully et al. (2019) developed a randomized, double-blind, controlled trial, where randomly assigned patients received caplacizumab (10-mg intravenous loading bolus, followed by 10 mg daily subcutaneously) or placebo during plasma exchange for 30 days [5]. The primary outcome was time to normalize the platelet count, with discontinuation of plasma exchanged. The study revealed that among patients with TTP, treatment with caplacizumab was associated with faster normalization of the platelet count, lower incidence of composite TTP-related death, and a lower rate of recurrence of TP during the trial than the placebo [8].

## Conclusions

Patients with TTP typically present with fever, thrombocytopenia, hemolytic anemia, renal dysfunction, and neurologic dysfunction; tachycardia, tachypnea, and chest pain may also be present. Accurate and prompt diagnosis of TTP in an urgent care setting is crucial. Without timely and effective treatment, TTP has a high mortality. Its diagnosis is challenging as its presentation involves multiple organ systems, and comorbid conditions can impede a timely and accurate diagnosis. Understanding more about the various presentations of TTP and challenges in its diagnosis, through case reports such as the one presented, can reduce patient morbidities and ultimately save lives.
